# A novel early risk assessment tool for detecting clinical outcomes in patients with heat-related illness (J-ERATO score): Development and validation in independent cohorts in Japan

**DOI:** 10.1371/journal.pone.0197032

**Published:** 2018-05-09

**Authors:** Kei Hayashida, Yutaka Kondo, Toru Hifumi, Junya Shimazaki, Yasutaka Oda, Shinichiro Shiraishi, Tatsuma Fukuda, Junichi Sasaki, Keiki Shimizu

**Affiliations:** 1 Department of Emergency and Critical Care Medicine, School of Medicine, Keio University, Tokyo, Japan; 2 Department of Anesthesia, Critical Care, and Pain Medicine, Massachusetts General Hospital/ Harvard Medical School, Boston, Massachusetts, United States of America; 3 Department of Emergency Medicine, Graduate School of Medicine, University of Ryukyus, Okinawa, Japan; 4 Emergency Medical Center, Kagawa University Hospital, Kagawa, Japan; 5 Advanced Medical Emergency and Critical Care Center, Osaka University Hospital, Osaka, Japan; 6 Advanced Medical Emergency and Critical Care Center, Yamaguchi University School of Medicine, Yamaguchi, Japan; 7 Emergency and Critical Care Center, Aizu Chuo Hospital, Fukushima, Japan; 8 Emergency and Critical Care Center, Tokyo Metropolitan Tama Medical Center, Tokyo, Japan; Universidad Miguel Hernandez de Elche, SPAIN

## Abstract

**Background:**

We sought to develop a novel risk assessment tool to predict the clinical outcomes after heat-related illness.

**Methods:**

Prospective, multicenter observational study. Patients who transferred to emergency hospitals in Japan with heat-related illness were registered. The sample was divided into two parts: 60% to construct the score and 40% to validate it. A binary logistic regression model was used to predict hospital admission as a primary outcome. The resulting model was transformed into a scoring system.

**Results:**

A total of 3,001 eligible patients were analyzed. There was no difference in variables between development and validation cohorts. Based on the result of a logistic regression model in the development phase (n = 1,805), the J-ERATO score was defined as the sum of the six binary components in the prehospital setting (respiratory rate≥22 /min, Glasgow coma scale<15, systolic blood pressure≤100 mmHg, heart rate≥100 bpm, body temperature≥38°C, and age≥65 y), for a total score ranging from 0 to 6. In the validation phase (n = 1,196), the score had excellent discrimination (C-statistic 0.84; 95% CI 0.79–0.89, p<0.0001) and calibration (P>0.2 by Hosmer-Lemeshow test). The observed proportion of hospital admission increased with increasing J-ERATO score (score = 0, 5.0%; score = 1, 15.0%; score = 2, 24.6%; score = 3, 38.6%; score = 4, 68.0%; score = 5, 85.2%; score = 6, 96.4%). Multivariate analyses showed that the J-ERATO score was an independent positive predictor of hospital admission (adjusted OR, 2.43; 95% CI, 2.06–2.87; P<0.001), intensive care unit (ICU) admission (3.73; 2.95–4.72; P<0.001) and in-hospital mortality (1.65; 1.18–2.32; P = 0.004).

**Conclusions:**

The J-ERATO score is simply assessed and can facilitate the identification of patients with higher risk of heat-related hospitalization. This scoring system is also significantly associated with the higher likelihood of ICU admission and in-hospital mortality after heat-related hospitalization.

## Introduction

Exposure to high ambient temperatures can be deadly, making extreme summer heat a serious global public health threat [1,2,3]. The association of extreme summer heat with excess mortality and morbidity has been well documented in the last decades. For example, the unprecedented heat waves resulted in 700 excess death in Chicago in 1995 [4] and 14,800 excess deaths in France in 2003 [5,6]. While evidence suggests the adverse impacts of heat waves on human health in many regions [7], the morbidity and mortality related to heat condition are reduced by appropriate preventions and treatments [8].

Heat-related illnesses occur when high ambient temperatures overcome the body’s ability to dissipate heat. Sepsis and heatstroke have similar mechanisms and include the production and release of several pro-inflammatory cytokines in association with the systemic inflammatory response syndrome [9,10,11,12]. With the rapidly aging population in Japan, the heat-related hospitalization and mortality have emerged as a serious social problem in the recent years [13,14]. Concurrently, an increasing number of heat-related ambulance dispatches is reported every year [15]. Indeed, the growing incidence of heat-related disorders warrants early recognition of clinical risks in patients with heat illness. Thus, developing a useful assessment tool to assess the negative health impacts of heat waves is an unmet medical need. However, clinical studies with a large cohort, which investigated an early warning system to assess the outcomes of heat illness, are extremely limited.

We sought to establish a simple scoring system that can be useful for predicting the clinical outcomes of heat illness in the prehospital setting. The aim of this study to develop the novel prediction system for clinical outcomes after heat illness using the database from a large, multicenter observational registry of patients with heat illness in Japan. The outcomes of interest were hospital admission, ICU admission, and in-hospital mortality. Here, we present a novel scoring system, called the Early Risk Assessment Tool for Detecting Clinical Outcomes in Patients with Heat-related Illness (J-ERATO score). It is assessed using the prehospital six binary components to identify the risk of clinical deterioration in patients with heat-related illness.

## Materials and methods

### Study design and settings

An ad-hoc analysis was conducted using a registered database of the prospective, multicenter, observational study (Heatstroke STUDY 2010 and 2012) in Japan. Briefly, the Japanese Association for Acute Medicine (Heatstroke Surveillance Committee) established the Heatstroke STUDY in 2006. This study involved a survey of patients with presumed heat-related illness transferred to an emergency hospital by emergency medical services (EMS) and was conducted every two years beginning 2006. A total of 94 and 103 emergency hospitals from all over Japan were enrolled in the Heatstroke STUDY between June 1 and August 31, 2010 and between July 1, 2012 and September 30, 2012, respectively. Diagnosis as the heat-related illness was performed after patient’s ED arrival by each attending physician. The data was manually recorded by a staff at each participating hospital using specific record sheets. The study protocol was approved by the institutional review board or ethics committee at each participating medical institution (Japanese Red Cross Kitami Hospital, Teine Keijinkai Hospital, Sapporo City General Hospital, National Hospital Organization Hokkaido Medical Center, Hachinohe City Hospital, Iwate Medical University Hospital, Ishinomaki Red Cross Hospital, Tohoku University Hospital, Osaki Citizen Hospital, Akita University Hospital, Akita Red Cross Hospital, Yamagata Prefectural Central Hospital, Yamagata University Hospital, National Hospital Organization Mito Medical Center, Mito Saiseikai General Hospital, Tsukuba Medical Center Hospital, University of Tsukuba Hospital, Dokkyo Medical University Hospital, Dokkyo Medical University Nikko Medical Center, Isesaki Municipal Hospital, Maebashi Red Cross Hospital, Gunma University Hospital, Saitama Medical University Hospital, Dokkyo Medical University Koshigaya Hospital, National Defence Medical College Hospital, Kawaguchi Municipal Medical Center, Saitama Medical Center, Saitama Medical Center Jichi Medical University, Chiba University Hospital, Juntendo University Urayasu Hospital, Matsudo City Hospital, Kimitsu Chuo Hospital, Nippon Medical School Chiba Hokusoh Hospital, Chiba Emergency Medical Center, National Hospital Organization Disaster Medical Center, Nihon University Hospital, Tokai University Hachioji Hospital, Tokyo Medical University Hachioji Medical Center, Toho University Ohashi Medical Center, Tokyo Metropolitan Hiroo General Hospital, Nippon Medical School Tama Nagayama Hospital, Japanese Red Cross Medical Center, Kyorin University Hospital, St. Luke's International Hospital, Teikyo University Hospital, The Jikei University Hospital, Toho University Omori Medical Center, Nippon Medical School Hospital, Nihon University Itabashi Hospital, Ome Municipal General Hospital, Showa University Hospital, Tokyo Metropolitan Tama Medical Center, St. Marianna University. School of Medicine Fujisawa City Hospital, National Hospital Organization Yokohama Medical Center, Yokohama City University Medical Center, Tokai University Hospital, Saiseikai Yokohamashi Tobu Hospital, Kitasato University Hospital, Kawasaki Municipal Hospital, Nippon Medical School Musashi Kosugi Hospital, Yokohama City University Hospital, Yamanashi Prefectural Central Hospital, University of Yamanashi Hospital, Nagano Red Cross Hospital, Saku Central Hospital Advanced Care Center, Aizawa Hospital, Niigata City General Hospital, Niigata University Medical & Dental Hospital, Kanazawa University Hospital, Ishikawa Prefectural Central Hospital, Noto General Hospital, Kanazawa Medical University Hospital, Gifu Prefectural General Medical Center, Gifu University Hospital, Takayama Red Cross Hospital, Gifu Prefectural Tajimi Hospital, Chuno Kosei Hospital, Shizuoka Saiseikai General Hospital, Numazu City Hospital, Seirei Mikatahara General Hospital, Seirei Hamamatsu General Hospital, Hamamatsu University School of Medicine, Ichinomiya Municipal Hospital, Daiyukai General Hospital, TOYOTA Memorial Hospital, Nagoya City University Hospital, Aichi Medical University Hospital, Okazaki City Hospital, Chukyo Hospital, Handa City Hospital, Hamamatsu Medical Center, Ise Red Cross Hospital.

Mie Prefectural General Medical Center, Saiseikai Shigaken Hospital, Nagahama Red Cross Hospital, Japanese Red Cross Otsu Hospital, Japanese Red Cross Society Kyoto Daini Hospital, Osaka University Hospital, Osaka Mishima Emergency Critical Care Center, National Hospital Organization Osaka National Hospital, Osaka Prefectural Nakakawachi Medical Center of Acute Medicine, Kansai Medical University Medical Center, Kindai University Hospital, Osaka General Medical Center, Osaka Medical College Hospital, Kobe University Hospital, Hyogo Emergency Medical Center, Hyogo Prefectural Kakogawa Medical Center, Kakogawa West City Hospital, Hyogo Prefectural Nishinomiya Hospital, Shimane Prefectural Central Hospital, Nara Medical University, Japanese Red Cross Wakayama Medical Center, Wakayama Medical University Hospital, Tottori University Hospital, Kawasaki Medical School Hospital, Tsuyama Chuo Hospital, National Hospital Organization Kure Medical Center, Fukuyama City Hospital, Hiroshima Prefectural Hospital, National Hospital Organization Kanmon Medical Center, Tokuyama Central Hospital, Yamaguchi University Hospital, Tokushima Red Cross Hospital, Kagawa Prefectural Central Hospital, Kitakyushu City Yahata Hospital, St.Mary's Hospital, Fukuoka University Hospital, Kurume University Hospital, Saga University Hospital, Nagasaki University Hospital, Kumamoto Red Cross Hospital, National Hospital Organization Kumamoto Medical Center, Saiseikai Kumamoto Hospital, Naha City Hospital, Nanbu Medical Center/Nanbu Child Medical Center, Urasoe General Hospital), which waived the requirement for informed patient consent to ensure participant anonymity. This study was also approved by the institutional review board or ethics committee at Keio University Hospital (IRB approval No. 20120230).

### Patient selection

All adult patients enrolled in the Heatstroke STUDY 2010 and 2012 were included in this study. Patients who suffered out-of-hospital cardiac arrest were excluded arrest (systolic blood pressure [SBP] = 0 or ≤ 10 mmHg, heart rate = 0 bpm, respiratory rate = 0). In addition, patients with missing data on hospital admission were excluded.

Before to any modeling, we randomly selected 60% of the eligible patients for model development. The remaining 40% of patients were reserved as a validation cohort after model development. Thus, 1,805 and 1,196 patients were assigned in the development and validation cohorts, respectively.

### Data collection

The patients’ demographics, prehospital information collected by the EMS, and in-hospital information were prospectively recorded; these included weather of the day, incident location, preexisting functional dependency, age, sex, prehospital vital signs, past medical history (preexisting hypertension, heart disease, psychological disorder, diabetes mellitus, cerebrovascular disease, dementia, Parkinson’s disease, chronic kidney disease), symptoms assessed by the EMS at the scene (if patients had any abdominal conditions, such as abdominal cramps, nausea, vomiting and diarrhea, those patients were defined as having abdominal symptoms. Similarly, if patients had any muscular conditions, such as muscle cramps, spasms and pain, those patients were defined as having muscular symptoms), vital signs on ED arrival, laboratory data on arrival (white blood cell count, hematocrit, platelet count, blood urea nitrogen [BUN], creatinine, aspartate transaminase [AST], alanine transaminase [ALT], creatinine kinase [CK], C-reactive protein [CRP]), hospital admission, ICU admission, and survival to hospital discharge.

### Definition of outcomes

The primary outcome was hospital admission. Secondary outcomes were ICU admission and in-hospital mortality. Outcomes were assessed by the patient’s attending physicians.

### Sample size calculation

As we used a logistic regression model to construct a predictive score, the sample size had to be based on the events-per-variable ratio. This ratio had to be greater than 10. We had 710 and 459 events (patients with hospital admission) in the development and validation cohort, respectively. Therefore, we could construct a predictive model with 71 and 45 explanatory variables in the development and validation cohort, respectively [16].

### Score development

In the development phase, the scoring system was created. Considering the abundant evidences that the pathophysiology of heat illness has many similarities with the sepsis syndrome [9,10,11,12], we referred to the quick sequential organ failure assessment (qSOFA) score which is a method to estimate the risk of inpatient mortality in patients with suspected infection outside the ICU [17,18] and in the ED [19,20]. However, previous researches reported the poor accuracy of qSOFA for prehospital identification of severe sepsis [21] and some components of this score do not consider specific features of heat illness. Patients were divided into two categories with the following variables: age (defined as ≥65 years based on the previous evidence [22,23,24]), body temperature and prehospital HR (based on the median values [38°C and 100 bpm, respectively]), and three components of qSOFA (prehospital SBP, RR and mental status) [19]. Multivariate logistic regression model was fit using the primary endpoint as a dependent variable. A set of potential confounders included the binary categories of elderly age (< 65 or ≥ 65 y), prehospital body temperature (< 38 or ≥ 38°C), prehospital HR (< 100 or ≥ 100 bpm), prehospital RR (< 22 or ≥ 22 /min)[19], prehospital SBP (> 100 or ≤ 100 mmHg)[19], and prehospital mental status (GCS = 15 or <15)[19], year of incidence, weather of the day, incident location, preexisting functional dependency, sex, past medical histories, and whether or not patients had abdominal or muscular symptoms at prehospital setting. Thereafter, based on the results, biological plausibility [21,25], and the previous knowledge [22,23,24], the J-ERATO score was defined. Favoring simplicity over accuracy, a point score of 1 was assigned to each variable in the final model, irrespective of the regression coefficients [18].

### Score validation

In the validation phase, logistic regression was used to analyze the prediction model. Discrimination and calibration were assessed by the C-statics and Hosmer-Lemeshow tests to indicate risk score performance. On the other hand, no calculations were made of the classical indicators of a diagnostic test, such as sensitivity, specificity, predictive values and likelihood ratios, as the test constructed does not indicate a single value (positive or negative) but rather a probability of hospital admission associated with each score. Accordingly, differences were studied between the expected (given by the predictive model) and the observed events to determine whether the reality corresponded to what was indicated by the model. A similar methodology has been used in other studies [26].

### Statistical analysis

Categorical variables are presented as the number (frequency), while continuous variables are presented as the median (interquartile range) because the duration of all continuous variables in our data showed non-normal distribution. The differences between the groups were tested with Kruskal-Wallis test. The chi-square or Fischer’s exact test was used to compare binary variables. To improve the quality of analyses, a multiple imputation was performed to replace each non-outcome missing value with a set of substituted plausible values by creating five filling-in copies to reduce bias caused by incomplete data [27,28]. Multivariate logistic regression models were constructed in each imputed copy, and the results of the five imputed copies were combined into one model, from which the statistical inference was taken [28,29].

All P values are two-tailed, and a P < 0.05 was considered significant. The outcome odds ratio was reported along with the P-values and 95% CIs. Statistical analyses were performed using IBM SPSS Statistics for Windows, Version 23.0 (IBM Corp., Armonk, NY).

## Results

### General characteristics

**Fig 1:**
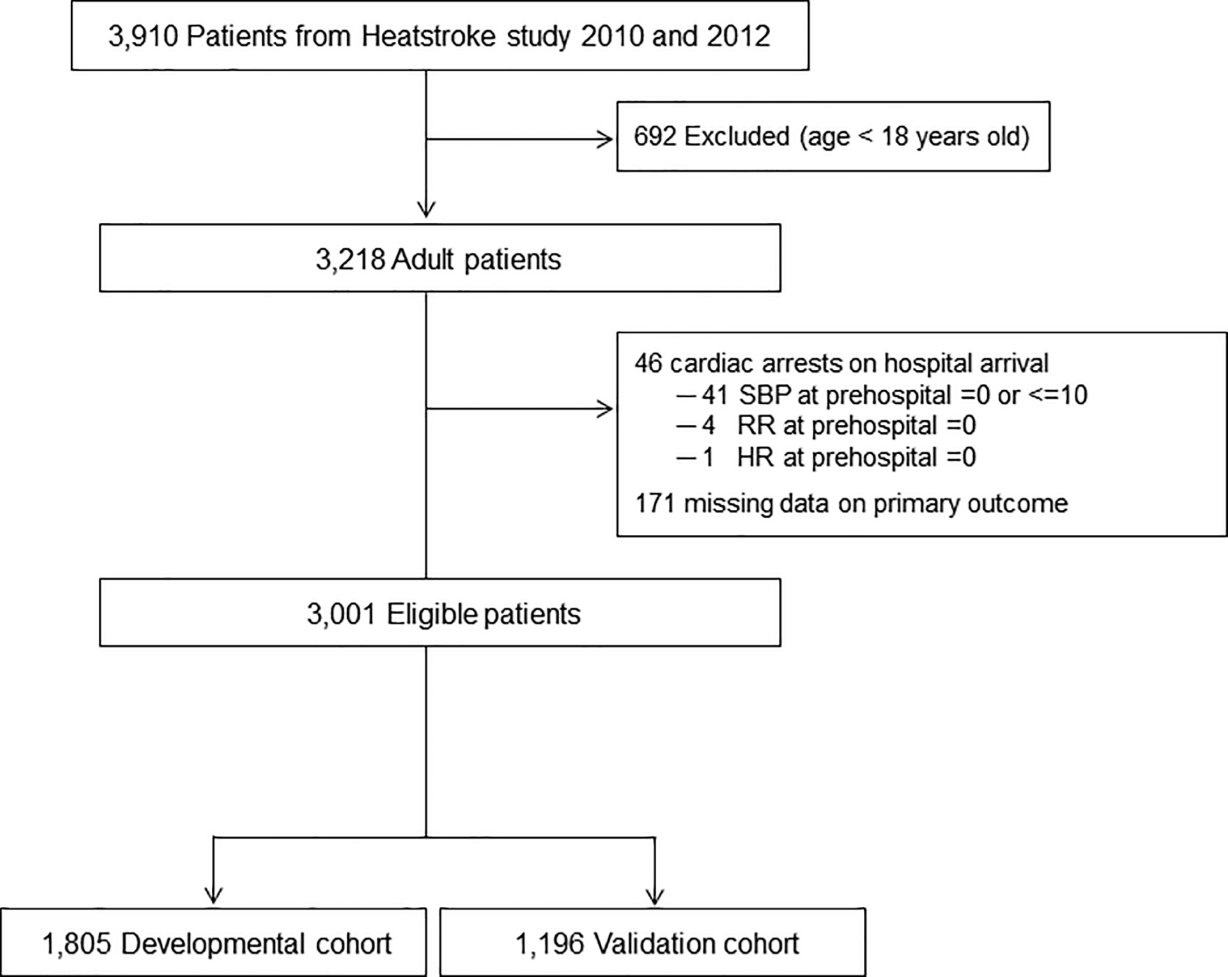
Patient selection.

In total, 3,910 patients who were transferred to the 172 hospitals were consecutively enrolled in the Heatstroke STUDY 2010 (between June 1, 2010, and August 31, 2010) and 2012 (between July 1, 2012, and September 30, 2012). After the exclusion of patients based on the predetermined criteria, 3,001 eligible patients were included. Of those, 1,805 and 1,196 patients were assigned in the development and validation cohorts, respectively (**Fig 1**).

**Table 1** shows the information for each cohort. There were 710 (39.3%) and 459 (38.4%) cases of hospital admission in the development and validation cohorts, respectively. There was no significant difference in variables between each group.

**Table 1:** Heat-related illness patients’ demographics, prehospital findings, and event characteristics between development and validation cohorts.

Variable	Development cohort (n = 1,805)		Validation cohort (n = 1,196)	P-value	
Weather				0.157	
Sunny	1388 (76.9)		953 (79.7)		
Cloudy	99 (5.5)		50 (4.2)		
Rainy	3 (0.2)		4 (0.1)		
Missing	315 (17.5)		189 (15.8)		
Location				0.078	
Outdoor	954 (52.9)		643 (53.8)		
Indoor	79 (4.4)		72 (6.0)		
Missing	772 (42.8)		481 (40.2)		
Functional dependency				0.176	
Not disabled	1399 (46.6)		950 (79.4)		
Disabled	195 (10.8)		132 (11.0)		
Missing	211 (11.7)		114 (9.5)		
Male sex	1257 (69.6)		834 (69.7)	0.390	
Missing	23 (1.3)		9 (0.8)		
Hypertension	332 (18.4)		211 (17.6)	0.628	
Heart disease	129 (7.1)		70 (5.9)	0.178	
Psychological disorder	120 (6.6)		79 (6.6)	1.0	
Diabetes mellitus	130 (7.2)		91 (7.6)	0.669	
Cerebrovascular disease		99 (5.5)	63 (5.3)	0.869	
Parkinson disease		16 (0.9)	20 (1.7)	0.060	
Chronic kidney disease		8 (0.4)	5 (0.4)	1.0	
Dementia	64 (3.5)		44 (3.7)		0.842
Age, years	56 (35−75)		55 (37−74)		0.478
Missing	5 (0.3)		1 (0.1)		
Abdominal symptom	232 (12.9)		151 (12.6)		0.403
Missing	781 (43.3)		491 (41.1)		
Muscular symptom	285 (15.8)		171 (14.3)		0.231
Missing	920 (51.0)		594 (49.7)		
Prehospital SBP, mmHg	126 (108−146)		125 (105−144)		0.685
Missing	701 (38.8)		458 (38.3)		
Prehospital RR, /min	24 (18−30)		24 (18−30)		0.545
Missing	892 (49.4)		591 (49.4)		
Prehospital BT, °C	37.7 (36.6−39.5)		37.6 (36.6−39.4)		0.761
Missing	780 (43.2)		496 (41.5)		
Prehospital HR, bpm	100 (82−120)		102 (85−120)		0.172
Missing	718 (39.8)		467 (39.0)		
Prehospital GCS category					0.618
GCS = 15	520 (28.8)		354 (29.6)		
GCS < 15	624 (34.6)		425 (35.5)		
Missing	661 (36.6)		417 (34.9)		
Hospital admission	710 (39.3)		459 (38.4)	0.619	

SBP = systolic blood pressure, RR = respiratory rate, GCS = Glasgow Coma Scale, BT = body temperature, ED = emergency department.

### Development of the J-ERATO score

**Fig 2:**
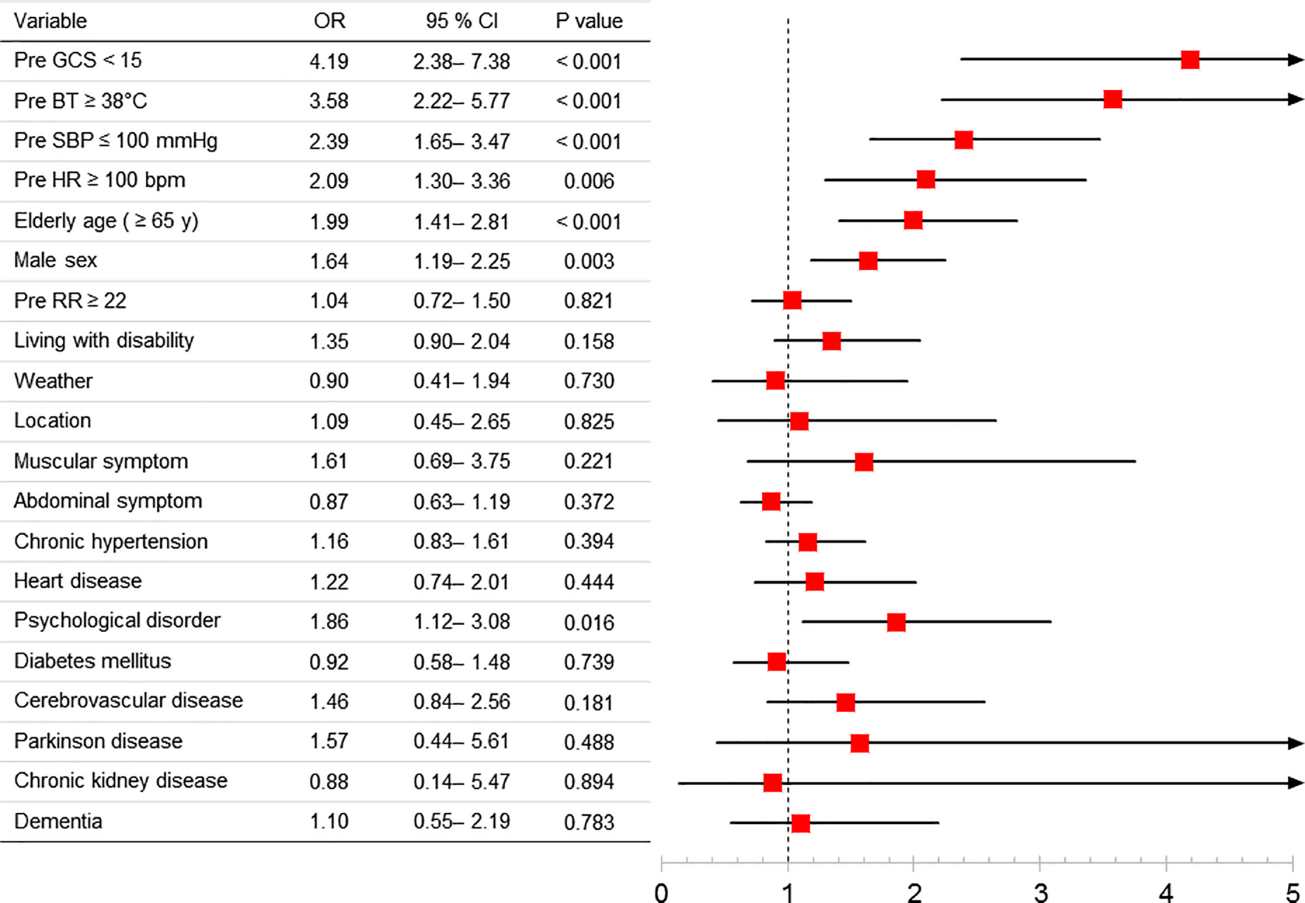
Logistic regression analysis for prediction of hospital admission in patients with heat-related illness.

A multivariate analysis showed that an increased likelihood of hospital admission was associated with prehospital GCS, prehospital body temperature, prehospital SBP, prehospital heart rate, age, male sex, and psychological disorder as past medical history (**Fig 2**). Based on the results and clinical plausibility, the J-ERATO score was defined as the total of the six binary components in the prehospital setting, including respiratory rate ≥ 22/min, altered mentation (Glasgow Coma Scale < 15), SBP ≤ 100 mmHg, prehospital HR ≥ 100 bpm, body temperature ≥ 38°C, and elderly age (≥ 65 years), for a total score ranging from 0 to 6.

### Validation of the J-ERATO score

**Fig 3:**
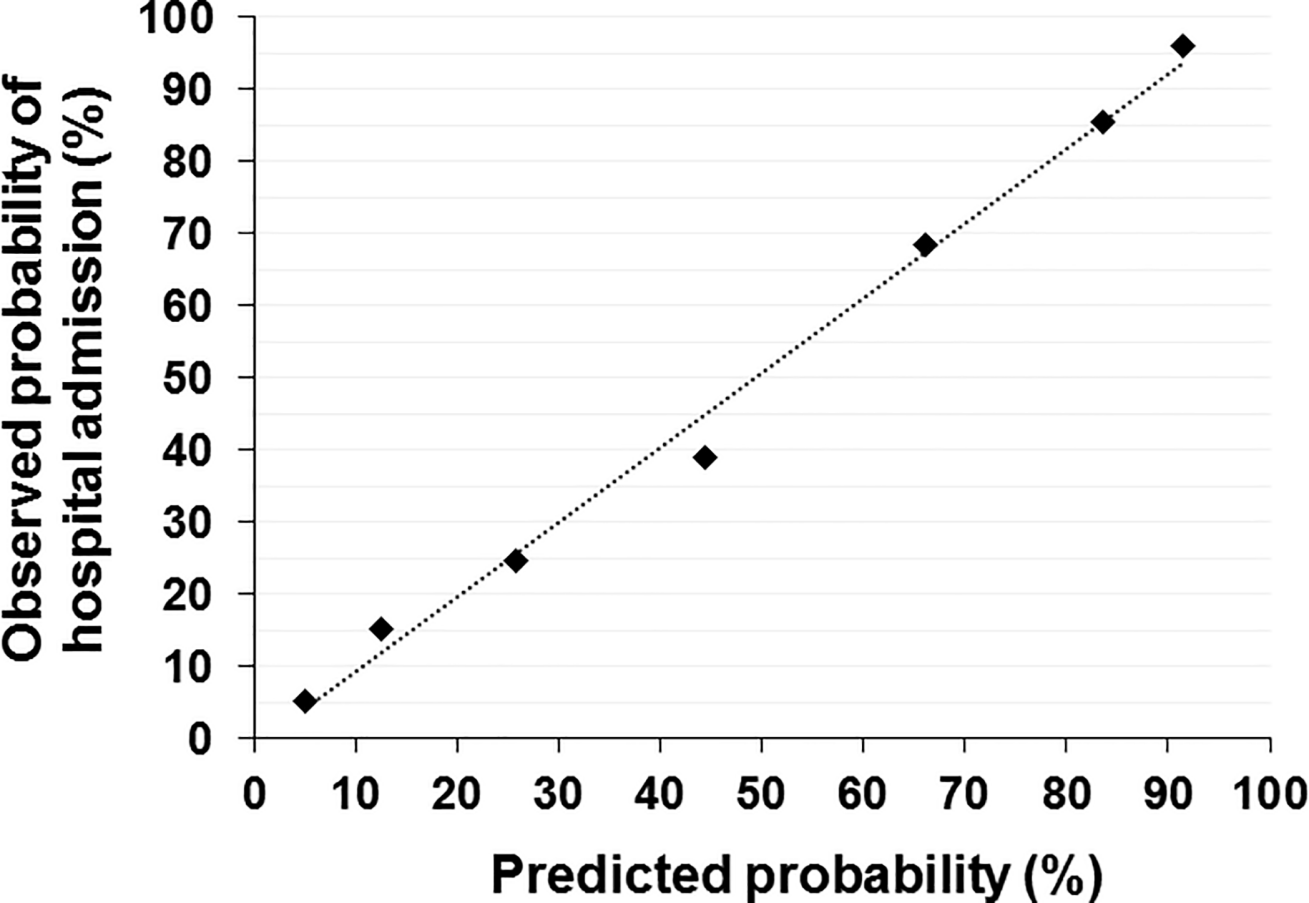
Calibration curve comparing the distribution of patients in relation to observed and estimated probability using logistic regression.

**Fig 4:**
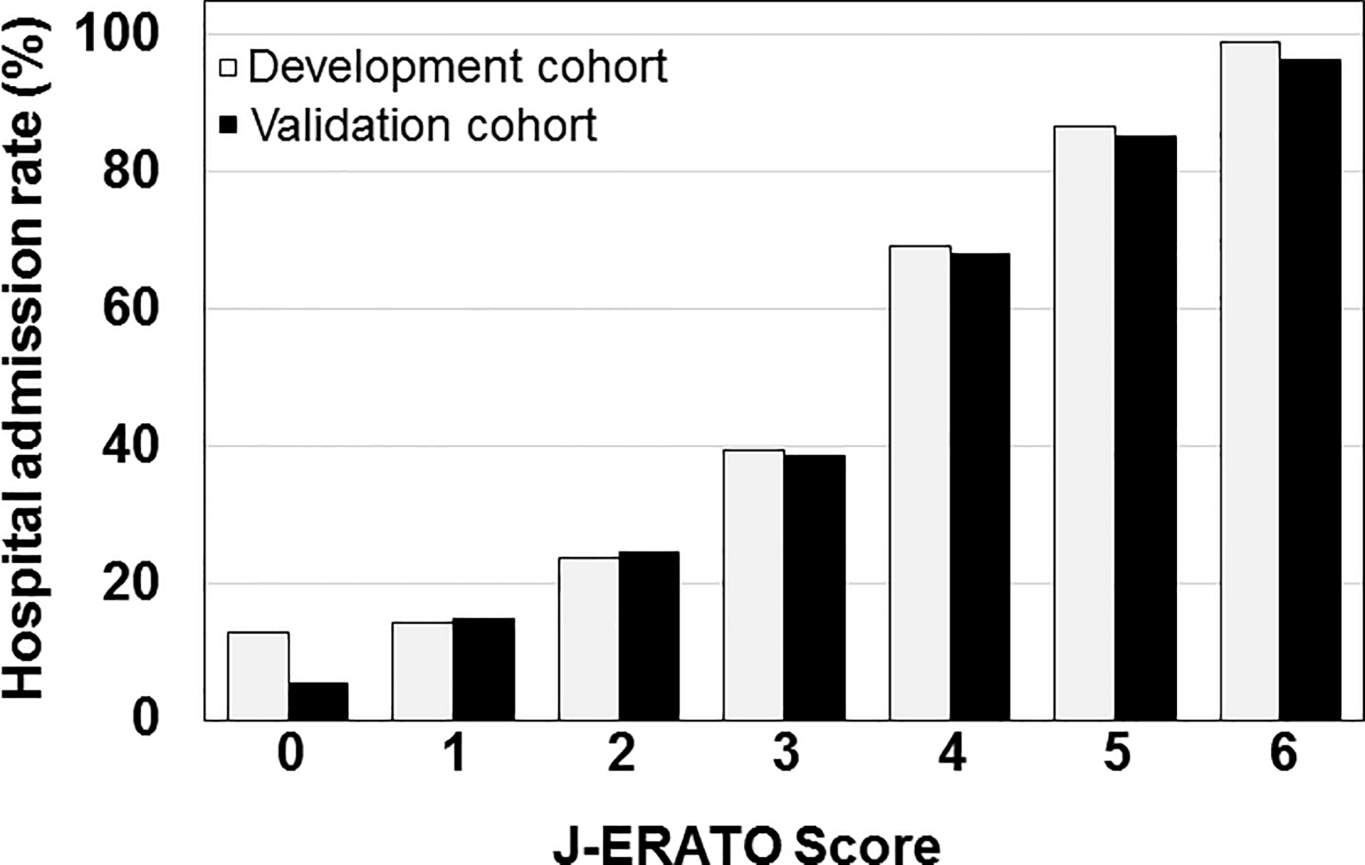
Proportions for hospital admission in the development and validation cohorts.

Patients’ clinical findings and laboratory data at ED arrival according to J-ERATO score in the validation cohort were shown in **Table 2**. The J-ERATO score had excellent discrimination (C-statistic 0.84; 95% CI 0.79–0.89, p<0.0001). The Hosmer-Lemeshow goodness-of-fit statistics were P*>*0.20 in all imputed copies of validation cohort, indicating good calibration. Calibration plot comparing the distribution of patients in relation to observed and estimated probability using logistic regression is presented in **Fig 3**. In addition, proportions for hospital admission in the development and validation cohorts are presented in **Fig 4**. The observed proportion of hospital admission increased with increasing J-ERATO score (score = 0, 5.0%; score = 1, 15.0%; score = 2, 24.6%; score = 3, 38.6%; score = 4, 68.0%; score = 5, 85.2%; score = 6, 96.4%).

**Table 2:** Patients’ demographics and laboratory data on ED arrival according to J-ERATO score in the validation cohort.

Variable	Missing, n (%)	Low (≤ 1)	Medium (2–4)	High (≥ 5)	P-value
**Vital signs at ED arrival**					
SBP, mmHg	54 (4.5)	127 (117‒ 144)	128 (114‒ 148)	110 (83‒ 137)	0.001
HR, bpm	94 (7.9)	80 (67‒ 93)	95 (81‒ 116)	110 (83‒ 137)	< 0.001
GCS	34 (2.8)	15 (15‒ 15)	15 (12‒ 15)	7 (3‒ 13)	< 0.001
BT, °C	150 (12.5)	36.1 (36.7‒ 37.1)	37.5 (36.7‒ 38.9)	39.3 (38.4‒ 40.6)	< 0.001
**Laboratory data at ED arrival**					
White blood cell, × 10^3^/μL	93 (7.8)	7.9 (5.6‒ 11.6)	9.2 (6.6‒ 11.3)	9.1 (6.7‒ 10.4)	0.64
Hematocrit, %	115 (9.6)	41.8 (38.2‒ 46.6)	40.2 (36.2‒ 44.6)	38.5 (34.5‒ 43.4)	0.003
Platelet, ×10^4^/μL	112 (9.4)	22.0 (17.8‒ 27.1)	21.4 (15.7‒ 26.7)	16.9 (12.5‒ 25.2)	0.04
BUN, mg/dL	96 (8.0)	16.0 (11.1‒ 20.6)	19.0 (14.0‒ 25.1)	22.0 (16.1‒ 30.6)	0.001
Creatinine, mg/dL	87 (7.3)	0.9 (0.6‒ 1.2)	1.1 (0.8‒ 1.5)	1.4 (0.9‒ 1.9)	0.001
AST, U/L	97 (8.1)	25 (19‒ 33)	30 (21‒ 46)	38 (22‒ 110)	< 0.001
ALT, U/L	99 (8.3)	19 (14‒ 26)	22 (15‒ 35)	38 (19‒ 64)	< 0.001
CK, U/L	160 (13.4)	166 (98‒ 297)	182 (91‒ 526)	269 (124‒ 419)	0.015
CRP, mg/dL	173 (14.5)	0.1 (0.03‒ 0.40)	0.2 (0.06‒ 1.40)	0.3 (0.04‒ 0.81)	0.064

SBP = systolic blood pressure, RR = respiratory rate, GCS = Glasgow Coma Scale, BT = body temperature, ED = emergency department. P value for difference between groups by Kruskal-Wallis.

To further determine the association of the J-ERATO score with three outcomes, multivariate logistic regressions were conducted. Multivariate analyses showed that the J-ERATO score was an independent positive predictor of hospital admission (adjusted OR, 2.43; 95% CI, 2.06–2.87; P < 0.001), ICU admission (3.73; 2.95–4.72; P < 0.001) and in-hospital mortality (1.65; 1.18–2.32; P = 0.004)(**Table 3**), suggesting that J-ERATO score was associated with clinical outcomes with heat-related illness.

**Table 3:** Associations of the J-ERATO score with the outcomes in patients with heat-related illness.

Outcomes	n/ N	OR	95% CI	P-value
**Hospital admission**	459/ 1196			
Crude		2.43	2.14–2.76	< 0.001
Adjusted		2.43	2.06–2.87	< 0.001
**ICU admission**	165/ 991			
Crude		3.64	2.90–4.58	< 0.001
Adjusted		3.73	2.95–4.72	< 0.001
**Hospital mortality**	30/ 1106			
Crude		1.61	1.24–2.09	< 0.001
Adjusted		1.65	1.18–2.32	< 0.001

Logistic regression models were used with adjustment for year of incident, weather, location, preadmission functional status, sex, past medical history (preexisting hypertension, heart disease, psychological disorder, diabetes mellitus, cerebrovascular disease, dementia, Parkinson disease, chronic kidney disease), symptoms assessed by EMS at scene (presence of abdominal symptoms, presence of muscular symptoms).

## Discussion

Although heat-related hospitalization and mortality became a serious social problem worldwide, studies for the risk assessment tools predicting the occurrence of these problems after heat illness have been limited. The current study demonstrated that the novel J-ERATO score, which included only information regarding prehospital vital signs and age of patient, was independently associated with the increased likelihood of hospital admission from ED among adult patients with heat-related illness. The associations of higher J-ERATO scores with increased ICU admission rate and mortality were also observed. Because most of heat-illness patients access acute emergency care through 1-1-9 EMS in Japan, optimizing field triage has been a crucial aspect of concentrating high-need patients in the hospitals most capable of caring for them. Thus, J-ERATO score can be helpful for EMS personnel to triage patients triage and choose an appropriate hospital.

Although several studies have identified the risk factors for mortality in patients with heatstroke admitted at the ICU [6,30,31], the studies focusing on early screening tools predicting the clinical outcomes in patients with heat-related illness in the prehospital setting do not exist. The J-ERATO score was developed based on the results of multivariate logistic model in the developmental cohort. The score included the six binary components such as heart rate, body temperature and age in addition to the three components of qSOFA score in the prehospital setting. Previous evidence showed that older adults are more prone to heat stress due to an existing chronic medical condition that changes their normal body responses to heat and takes prescription medicines that affect the body’s ability to control its temperature or sweat [21,22,23,24,25]. A study in the USA reported that extreme heat wave was associated with a 3% increase in all-cause hospitalization in older people [22]. The qSOFA score was recently derived by a panel of experts as a scoring system for patients with suspected sepsis [17]. The utility of the qSOFA to the estimate risk of inpatient mortality was validated in patients with suspected infection outside the ICU [18] and in adult ED patients likely to be admitted both with and without suspected infection [20], whereas qSOFA had poor sensitivity for prehospital identification of severe sepsis and septic shock [32]. In addition, since our data showed that the higher J-ERATO score was significantly associated with the poorer vital sings at ED arrival and worsened laboratory data (**Table 2**), the score in the prehospital field can predict the physical condition. Taken together, the components included in the J-ERATO score can be considered to be well reflected the physiological aspects of patients with acute illness.

Using the prehospital binary parameters, patients with hospital admission were well differentiated from those with ED discharge to home, patients with ICU admission from those admitted to general ward, and non-survivors from survivors after heat illness. Our findings assist the ED in the risk stratification of patients with heat illness. Moreover, the advantage of prognostication using J-ERATO is that it can be readily performed at scene by EMS personnel, which has a significant clinical benefit. Thus, it is conceivable that clinical usage of the J-ERATO score by EMS in the field can improve the outcomes among those patients.

Evidence has shown that having a pre-existing psychiatric illness can more than triple the risk of death during a heat wave [33]. Previous studies have demonstrated that several mental illness and/or behavioral disorder hospitalizations are associated with concurrent diagnoses of hospitalizations for a heat-related illness [34, 35]. Recent study also suggested an increased risk of hospitalization among diagnoses of psychoses with concurrent heat-related illness, including dementia and schizophrenia, which may have similar symptoms of cognitive impairment as those with psychoactive substance abuse [35]. A contributing factor for hospitalizations after a heat-related illness in psychotic patients may be due to medications that are used for several mental illnesses and other chronic conditions. Many medications used in psychotic patients increase vulnerability to heat-related morbidity by altering the body’s ability to thermoregulate. These types of medications have been suggested as one of the causes for heat-related hospitalizations and mortality [36]. Consistent with these, the logistic regression model in the development phase revealed that pre-existing psychotic disorder was associated with hospitalization. Considering that it is challenging for EMS personnel to obtain the certain information regarding past medical history from patients with altered mentation, we did not include the past medical history as a component of the predictive score. Of note, the J-ERATO score was an independent positive predictor of the outcomes after heat illness after accounting for the past medical histories including psychotic disorder.

Our study had several limitations. First, since this is an observational study, the positive association between J-ERATO scores and worsened clinical outcomes does not necessarily prove the causality and can be confounded by unmeasured factors. Other variables may have contributed, which were unable to control or that were not collected a priori. Second, a multivariate analysis to assess the association between J-ERATO score and mortality was not conducted because of the small numbers of events. Thus, further investigations to determine the role of J-ERATO on mortality after heat illness are warranted. Third, the treatments patients received during ED visits and after admission were not controlled in this study. Examples of potential confounding variables include intravenous fluid therapy, cooling methods, time interval from ED arrival to ED discharge, and post-admission care such as ventilation, renal replacement therapy, and active cooling device for heat stroke. Finally, this large, multicenter cohort study describes that in Japan only. In fact, Japan has the highest proportion of elderly persons who are more prone to heat-related health problems. Thus, similar studies with data from other countries may result in different findings.

## Conclusion

The J-ERATO score is easily and quickly calculated before ED arrival. Higher J-ERATO score was associated with higher likelihood of hospital admission, ICU admission, and increased mortality and was independent positive predictor of hospitalization among patients with the heat-related illness. Further clinical studies are warranted to validate J-ERATO score as a useful tool in the prehospital setting to rapidly assess the potential for adverse outcomes in heat illness. Early recognition of risk likelihood using the J-ERATO score helps in the early allocation of resources, for example, the need for intensive treatments for patients with higher J-ERATO scores.

## Supporting information

S1 Table Deidentified dataset used for the statistical analyses. Data_de_identified.
(XLSX)
